# Two cases of a perforated duodenal diverticulum after gastrectomy with Roux-en-Y reconstruction

**DOI:** 10.1186/s40792-019-0738-y

**Published:** 2019-11-05

**Authors:** Shusuke Yagi, Satoshi Ida, Manabu Ohashi, Koshi Kumagai, Naoki Hiki, Takeshi Sano, Souya Nunobe

**Affiliations:** 1 Department of Gastroenterological Surgery, Cancer Institute Hospital of Japanese Foundation for Cancer Research, 3-8-31, Ariake, Koto, Tokyo, 135-8550 Japan; 2 Department of Upper Gastrointestinal Surgery, Kitasato University School of Medicine, 1-15-1, Kitasato, Minami, Sagamihara, Kanagawa 252-0374 Japan

**Keywords:** Perforated duodenal diverticulum, Gastrectomy, Roux-en-Y reconstruction, Intraduodenal pressure, Duodenal diverticulum

## Abstract

**Background:**

What type of reconstruction procedure should be applied is one of the important issues in surgery for gastric cancer. We have several options for reconstruction procedure after distal gastrectomy. The Billroth II and Roux-en-Y reconstruction have a duodenal stump while the Billroth I does not have it, which is the biggest structural difference in these procedures. An increase in intraduodenal pressure due to the formation of duodenum stump occasionally causes severe complication such as duodenal stump leakage; however, a duodenal diverticulum perforation after the Roux-en-Y reconstruction has not yet been reported. Herein, we report two cases of a perforated duodenal diverticulum after gastrectomy with the Roux-en-Y reconstruction.

**Case presentation:**

The first case was a 66-year-old man who presented to our hospital with an acute onset right-upper-quadrant abdominal pain. He had undergone laparoscopic distal gastrectomy with the Roux-en-Y reconstruction for the early gastric cancer 15 months before. A large periampullary diverticulum had been detected during the checkup before the gastrectomy. Abdominal contrast-enhanced CT showed a retroperitoneal fluid collection with gas present at the second part of the duodenum. Therefore, a perforated duodenal diverticulum with abdominal abscess was diagnosed, and an emergency laparotomy was performed. Pancreaticoduodenectomy was performed because of severe duodenal inflammation and surrounding tissue damage. The second case was a 52-year-old man who had undergone open distal gastrectomy for locally advanced gastric cancer. Multiple non-ampullary duodenal diverticula had also been identified during the preoperative checkup. On the 2nd postoperative day, he presented with a sudden-onset abdominal pain with peritoneal irritation signs, and intestinal fluid was identified through the intraperitoneal drainage tube placed in a suprapancreatic site during his previous gastrectomy. Therefore, an emergency laparotomy was performed. During laparotomy, a perforated diverticulum at the second part of the duodenum was detected. The perforated duodenum diverticulum was directly sutured with drainage of the retroperitoneal space.

**Conclusions:**

It is necessary to recognize that the Roux-en-Y reconstruction after gastrectomy for gastric cancer patients with duodenal diverticulum might cause a perforation of the diverticulum.

## Background

What type of reconstruction procedure should be applied is one of the important issues in surgery for gastric cancer. We have several options for reconstruction procedure after distal gastrectomy, such as the Billroth I, Billroth II, and Roux-en-Y (R-Y) reconstruction. The Billroth II and R-Y reconstruction have a duodenal stump while Billroth I does not have it, which is the biggest structural difference in these procedures. We can choose any procedure in many cases, but we have to consider such a distinctive feature in a specific case.

Postoperative complications with the R-Y reconstruction particularly include Roux stasis syndrome, Petersen’s hernia, and duodenal stump leakage [1–4]. Duodenal stump leakage, which is well known as a life-threatening complication, is considered to be caused by increased pressure of the edge of the stump due to intestinal peristalsis [5, 6]. Furthermore, not only duodenal stump leakage but also perforation of duodenal diverticulum is reported after gastrectomy [7].

The duodenum is the second most common site, after the colon, for diverticulum in the alimentary tract. Secondary acquired pseudodiverticula, which only contain mucosal and serosal layers, are the most frequent type of duodenal diverticulum. They are pulsion diverticula resulting from a combination of the lack of muscle layer of the duodenum. Although a perforated duodenal diverticulum is rare, it is a serious and fatal complication [8, 9]. An occurrence of a perforated duodenal diverticulum after gastrectomy has been reported only in the Billroth II reconstruction. And it is considered that an increase in the pressure of duodenum due to the formation of duodenum stump might affect the perforation of a duodenal diverticulum, similar to the duodenal stump leakage [7].

So far, a duodenal diverticulum perforation after the R-Y reconstruction has not yet been reported. Herein, we report two cases of a perforated duodenal diverticulum after gastrectomy with the R-Y reconstruction.

## Case presentation

### Case 1

**Fig. 1:**
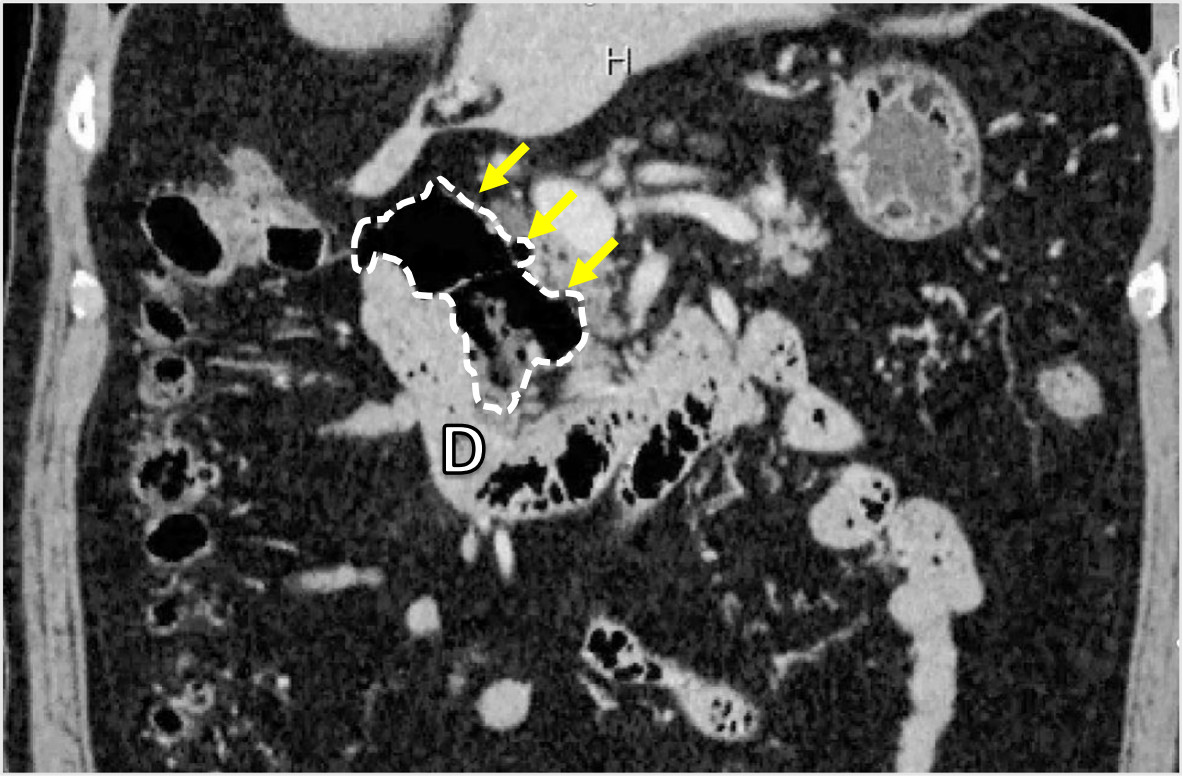
Computed tomography of case 1 before gastrectomy. A large periampullary diverticulum (yellow arrow) situated at the second part of the duodenum

A 66-year-old man presented to our emergency department with an acute onset of right-upper-quadrant abdominal pain. He had undergone laparoscopic distal gastrectomy with the R-Y reconstruction for the treatment of early gastric cancer 15 months before at our department. A large periampullary diverticulum was detected using contrast-enhanced computed tomography (CT) during the checkup before the gastrectomy (Fig. 1). The size of this duodenal diverticulum is 55 mm in length and 25 mm in width.

**Fig. 2:**
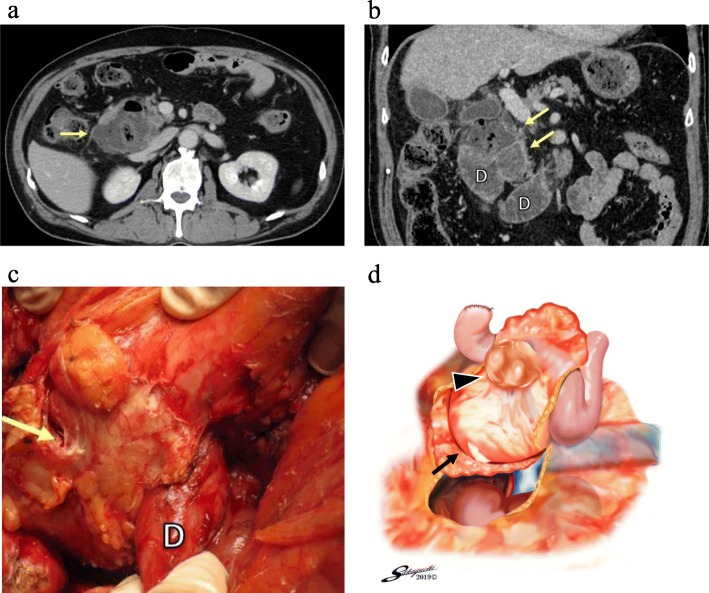
Computed tomography of case 1 with a perforated duodenal diverticulum and surgical findings. Abdominal contrast-enhanced CT (**a**, axial plane) showed an extraluminal fluid collection with gas (yellow arrow) surrounding the duodenum. Coronal plane (**b**) showed that an abscess (yellow arrows) was suspected around the second and third parts of the duodenum (D). A retroperitoneal abscess (yellow arrow) behind the second part of the duodenum (D) was found after performing Kocher maneuver (**c**). The schema (**d**) showed that a white-yellow retroperitoneal abscess (black arrow) was seen behind the perforated duodenal diverticulum (black arrowhead). And the right kidney was seen after performing Kocher maneuver

His vital signs on arrival were stable (body temperature, 35.7 °C; blood pressure, 157/79 mmHg; pulse rate, 64 beats/min). Physical examination revealed right-upper-quadrant abdominal pain without peritoneal irritation signs. Laboratory findings showed that his white blood cell (WBC) count was 15,000/μl, hemoglobin level was 10.0 g/dl, platelet count was 212,000/μl, and C-reactive protein (CRP) level was 12.0 mg/dl. Abdominal X-ray showed no intraperitoneal free air or sign of enteroparalysis, intestinal obstruction, and constipation. Abdominal contrast-enhanced CT showed a retroperitoneal fluid collection with gas present at the second part of the duodenum (Fig. 2a, b). Therefore, a perforated duodenal diverticulum with abdominal abscess was diagnosed.

Subsequently, an emergency laparotomy was performed. The duodenum was mobilized by Kocher maneuver, and an inflamed duodenal diverticulum with extensive retroperitoneal abscess was detected (Fig. 2c, d). Pancreaticoduodenectomy was performed because of severe duodenal inflammation and surrounding tissue damage. Histopathological examination revealed the presence of a perforated duodenal diverticulum with abscess. The postoperative course was uneventful, and the patient was discharged on the 23rd postoperative day.

### Case 2

**Fig. 3:**
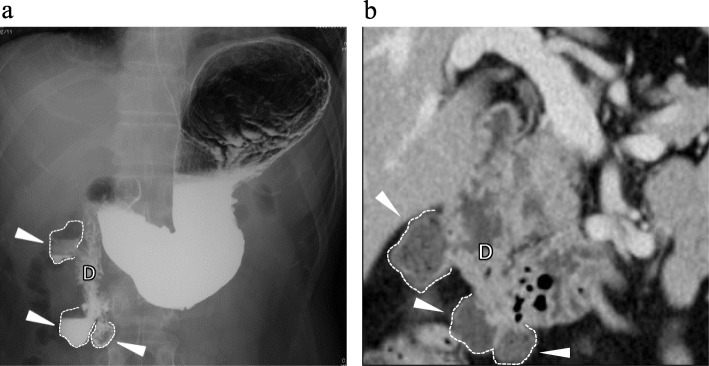
An upper gastrointestinal series and a contrast-enhanced CT of case 2 before gastrectomy. Multiple non-ampullary duodenal diverticula (white arrowhead) were detected at the second and third parts of the duodenum (D) (**a**, **b**)

A 52-year-old man underwent open distal gastrectomy with the R-Y reconstruction for locally advanced gastric cancer. An upper gastrointestinal series and a contrast-enhanced CT, which were preoperatively performed for gastric cancer, showed multiple non-ampullary duodenal diverticula located at the second and third parts of the duodenum (Fig. 3a, b). Each size of these diverticula is about 30 mm. On the 2nd postoperative day, he presented with a sudden-onset abdominal pain, and intestinal fluid was identified through the intraperitoneal drainage tube placed in a suprapancreatic site during his previous gastrectomy. He was afebrile with a blood pressure of 167/99 mmHg and a heart rate of 78 beats/min. Physical examination revealed the presence of right-upper-quadrant abdominal pain, with peritoneal irritation signs. Furthermore, laboratory tests showed a WBC count of 8300/μl, hemoglobin level of 14.5 g/dl, platelet count of 93,000/μl, and CRP level of 16.1 mg/dl.

**Fig. 4:**
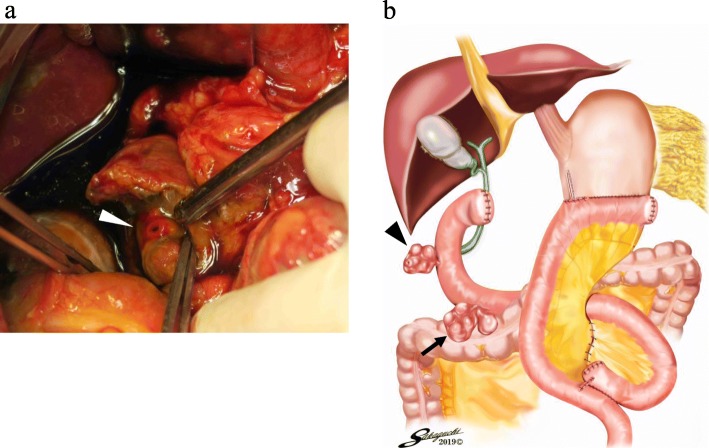
Surgical findings of case 2. A perforated duodenal diverticulum (white arrowhead) was found at the second part of the duodenum (**a**). The schema showed that a perforated duodenal diverticulum (black arrowhead) was seen at the second part of the duodenum. And the other duodenal diverticulum (black arrow) was detected at the third part of the duodenum, which was not perforated at the time of the emergency operation (**b**)

The presumptive diagnosis was a perforated duodenal diverticulum; therefore, an emergency laparotomy was performed. During laparotomy, a perforated diverticulum at the second part of the duodenum was detected and contaminated ascites were observed (Fig. 4a, b). There was no evidence of ileus, small bowel obstruction, or constipation that would raise intraduodenal pressure. The perforated duodenum diverticulum was directly sutured with drainage of the retroperitoneal space. Further, an intraluminal drainage tube was introduced via the jejunum in a retrograde fashion for duodenal decompression. Additionally, cholecystectomy was performed, and a biliary drainage tube was inserted into the duodenum through the cystic duct for drainage of the bile duct. Moreover, a jejunostomy tube was inserted for postoperative enteral nutrition.

On the 5th postoperative day, he developed an intra-abdominal abscess. Further, on the 15th postoperative day, another duodenal diverticulum located at the third part of the duodenum was perforated (Fig. 4b). This perforated duodenal diverticulum was treated with nonoperative management (drainage and fistulation). The patient’s condition improved with the drainage and antibiotic therapy, and he was discharged on the 53rd postoperative day.

## Discussion

Most duodenal diverticula are asymptomatic, with complications being reported in only 5–10% patients [9]. In addition, two thirds of the duodenal diverticula are found within 2.0 cm of the ampulla of Vater [10]. Major complications include obstruction of the biliary or pancreatic ducts, hemorrhage, and perforation due to diverticulitis [11, 12]. Thorson et al. have reviewed 162 cases with a perforated duodenum diverticulum [13]. The most common cause was found to be diverticulitis, which accounted for 62% of the 162 cases reviewed, followed by enterolithiasis (10%), iatrogenesis (5%), ulceration (5%), trauma (4%), and the presence of a foreign body (2%).

One case of a perforated duodenal diverticulum after gastrectomy followed by the Billroth II reconstruction has been reported [7]. An increase in intraduodenal pressure was suggested to be one of the causes of a perforated duodenal diverticulum. Additionally, 11 cases of a perforated duodenal diverticulum after gastrectomy have been reported in Japanese literature. And, all of them were reconstructed by the Billroth II method. Using manometry equipment, Filipovic et al. have reported that the intraluminal duodenal stump pressure after gastrectomy reconstructed by the Billroth II method, which forms a blind loop, was higher than that at the preoperative state [14]. Moreover, the longer afferent loop reportedly caused the increase in the intraduodenal stump pressure. To the best of our knowledge, the patients in this study are the first reported cases of a perforated duodenal diverticulum after gastrectomy with the R-Y reconstruction. Considering the difference between the length of an afferent jejunal loop for the Billroth II reconstruction and that for the R-Y reconstruction, the results of the duodenal pressure increase observed with the R-Y reconstruction might not be completely applicable to that with the Billroth II reconstruction. However, similar to the Billroth II reconstruction, the R-Y reconstruction also results in the formation of a stump in the duodenum; therefore, the intraduodenal pressure might increase. The excessive elevation of duodenal pressure may lead to a perforated duodenal diverticulum, as observed in our cases. Additionally, the sizes of the duodenal diverticulum of our two cases are relatively large, which might impact these clinical courses. Attention should especially be given to a perforated duodenal diverticulum due to excessive elevation of duodenal pressure when a duodenal diverticulum is large, because the wall of duodenal diverticulum is fragile.

Other causes of duodenal perforation include enterolithiasis, the presence of a foreign body, inflammation of the abdominal cavity, and iatrogenesis [13]. Reportedly, enterolithiasis associated with the Billroth II reconstruction is another cause of perforated duodenal diverticulum [7]. Tsukamoto et al. have reported the presence of a stone in the duodenum caused inflammation, and which resulted in a perforated duodenal diverticulum. However, enterolith and foreign body were not observed in the present cases. Moreover, inflammation of the abdominal cavity might cause duodenal perforation. However, in case 1, the presence of postoperative inflammation might not have resulted in the perforated duodenal diverticulum because gastrectomy had been performed > 1 year before and no signs of inflammation were detected. Although the perforated duodenal diverticulum occurred early in the postoperative period in case 2, pancreatic juice leakage or abscess formation was not observed before the onset. Additionally, the duodenal diverticulum might have been damaged during the previous gastrectomy. However, in case 1, the possibility of an iatrogenic cause is very low because Kocher maneuver to mobilize the duodenum retroperitoneally was not performed; thus, the duodenal diverticulum remained untouched. Also in case 2, it is unlikely that the duodenal diverticulum was damaged during operation, because another perforated duodenal diverticulum, which was located at the third part of the duodenum and was not touched during operation, occurred after the emergency laparotomy (Fig. 4b). Therefore, as previously mentioned, the perforated duodenal diverticulum in the present cases might be caused by the elevation of duodenal pressure.

For the treatment of perforated duodenal diverticulum, surgical intervention such as diverticulectomy with drainage or pancreaticoduodenectomy and nonsurgical treatment has been reported [13]. Surgical intervention is a common approach; however, the operative procedure is selected based on the condition of the perforated site and general condition of the patient [15–17]. In case 1, pancreaticoduodenectomy was performed because of a severe retroperitoneal inflammation, and in case 2, the perforated lesion was directly sutured with drainage of the retroperitoneal space and tube duodenostomy for duodenal decompression was performed.

Duodenum diverticula are usually incidentally found during esophagogastroduodenoscopy or an upper gastrointestinal series [9, 18, 19]. The presence of duodenal diverticulum should be carefully observed during preoperative examination for gastrectomy. In the present cases, duodenal diverticulum was detected before gastrectomy, but we failed to consider it as a serious complication; therefore, a surgical procedure was not devised. In other words, a perforated duodenal diverticulum might be prevented with the Billroth I or double-tract reconstruction, which does not result in duodenal stump formation. Thus, in case a duodenal diverticulum is detected, a reconstruction method without duodenal stump formation may effectively prevent the occurrence of a perforated duodenal diverticulum. Our two cases are so rare; however, a perforated duodenal diverticulum is a severe complication. Additionally, we can easily find out a duodenal diverticulum before gastrectomy because esophagogastroduodenoscopy or computed tomography is conducted during preoperative examination. Therefore, we propose to consider duodenal diverticulum as one of the factors for the decision of reconstruction procedure after gastrectomy.

## Conclusions

It is necessary to recognize that the R-Y reconstruction after gastrectomy for gastric cancer patients with duodenal diverticulum might cause a perforation of the diverticulum. Furthermore, the presence of duodenal diverticulum should be carefully observed by preoperative examination.

## Data Availability

The dataset supporting the conclusions of this article is available in the Springer Open.

## Abbreviations

CRP: C-reactive protein
CT: Computed tomography
R-Y: Roux-en-Y
WBC: White blood cell
